# Crystal structure of poly[tetra-μ-cyanido-ethanol­bis(2-iodo­pyrazine)­digold(I)iron(II)]

**DOI:** 10.1107/S2056989017014785

**Published:** 2017-10-24

**Authors:** Bin Fei, Olesia I. Kucheriv, Inna I. Tokmenko, Kateryna V. Terebilenko, Il’ya A. Gural’skiy

**Affiliations:** a School of Chemical Engineering, Nanjing University of Science and Technology, 210094 Nanjing, People’s Republic of China; b Department of Chemistry, Taras Shevchenko National University of Kyiv, Volodymyrska St. 64, Kyiv 01601, Ukraine; c Ukraine National O. O. Bogomoletz Medical University, 13 T. Shevchenko Blvd., Kyiv, Ukraine

**Keywords:** crystal structure, polymeric complex, di­cyano­aurate, iodo­pyrazine

## Abstract

In the title polymeric complex, [Au_2_Fe(CN)_4_(C_4_H_3_IN_2_)_2_(C_2_H_6_O)]_*n*_, the Fe^II^ cation is coordinated by two iodo­pyrazine mol­ecules, one ethanol mol­ecule and three di­cyano­aurate anions in a distorted N_5_O octa­hedral geometry. In the crystal, the di­cyano­aurate anions bridge the Fe^II^ cations to form polymeric chains propagating along the *b-*axis direction. Stabilization of the crystal structure is provided by O—H⋯N hydrogen bonds and π–π stacking between parallel iodo­pyrazine rings of neighbouring chains, the centroid–centroid distances being 3.654 (10) and 3.658 (9) Å.

## Abstract 2

The di­cyano­aurate anions bridge the Fe^II^ cations to form polymeric chains propagating along the *b* axis.

## Chemical context   

Among all coordination compounds, cyanide-based complexes attract considerable attention. The cyanide group can be coordinated in either a monodentate or bridging way, connecting different metal ions, leading to the formation of one-, two- or three-dimensional frameworks. The variety of possible structures of cyanide-based complexes results in a variety of functional properties for these coordination materials, such as the ability to include small guest mol­ecules (Klausmeyer *et al.*, 1998 ▸), act as room-temperature magnets (Garde *et al.*, 2002 ▸), display photomagnetic and magneto-optical properties (Mizuno *et al.*, 2000 ▸; Mercurol *et al.*, 2010 ▸), *etc*. The most representative examples of cyanide-bridged complexes are Prussian blue analogues, which form three-dimensional frameworks with general formula *A* ^I^ *M*_A_^II^[*M*_B_^III^(CN)_6_] (*A* = alkali ion, *M*_A_ and *M*_B_ = transition metal ions; Keggin & Miles, 1936 ▸). Prussian blue analogues are very attractive because of their facile synthesis and the possibility to manipulate the magnetic ordering of the material by selecting appropriate spin sources (Ohkoshi *et al.*, 1997 ▸).

Cyano­metallate complexes are typically characterized by a low-spin state of the metal ions; however, the introduction of a complementary ligand with weak ligand field strength can lead to the formation of spin-crossover compounds. This type of compound is mostly represented by Hofmann clathrate analogues with general formula [*M*(*L*)_*x*_{*M*′(CN)_4_}] where *M* = Fe^2+^, Co^2+^, Ni^2+^, Cu^2+^, Zn^2+^, Cd^2+^ and Mn^2+^, *M*′ = Ni^2+^, Pd^2+^, Pt^2+^ and *L* is either a unidentate or bridging ligand. The first compound of this type reported by Hofmann & Höchtlen (1903 ▸) was the [Ni(NH_3_)_2_{Ni(CN)_4_}] clathrate, which is able to incorporate benzene or other aromatic mol­ecules. In this structure, the bridging tetra­cyano­nikelate anions contribute to the formation of infinite layers that propagate in the *ab* plane (Powell & Rayner, 1949 ▸). However, the first Hofmann-clathrate analogue displaying spin-crossover behavior was [Fe(py)_2_{Ni(CN)_4_}] (Kitazawa *et al.*, 1996 ▸). Later, different examples have been obtained for the modification of the original Hofmann clathrates, notably with di- or octa­cyano­metallates (Gural’skiy *et al.*, 2016*b* ▸; Wei *et al.*, 2016 ▸). Another modification method is the use of different organic ligands; for example, the inclusion of a bidentate ligand such as pyrazine leads to the formation of a three-dimensional network (Niel *et al.*, 2001 ▸). Here we report a new cyanide-based compound with general formula [Fe(Ipz)_2_(EtOH){Au(CN)_2_}_2_] in which the Fe^II^ ions are stabilized in the high-spin state.
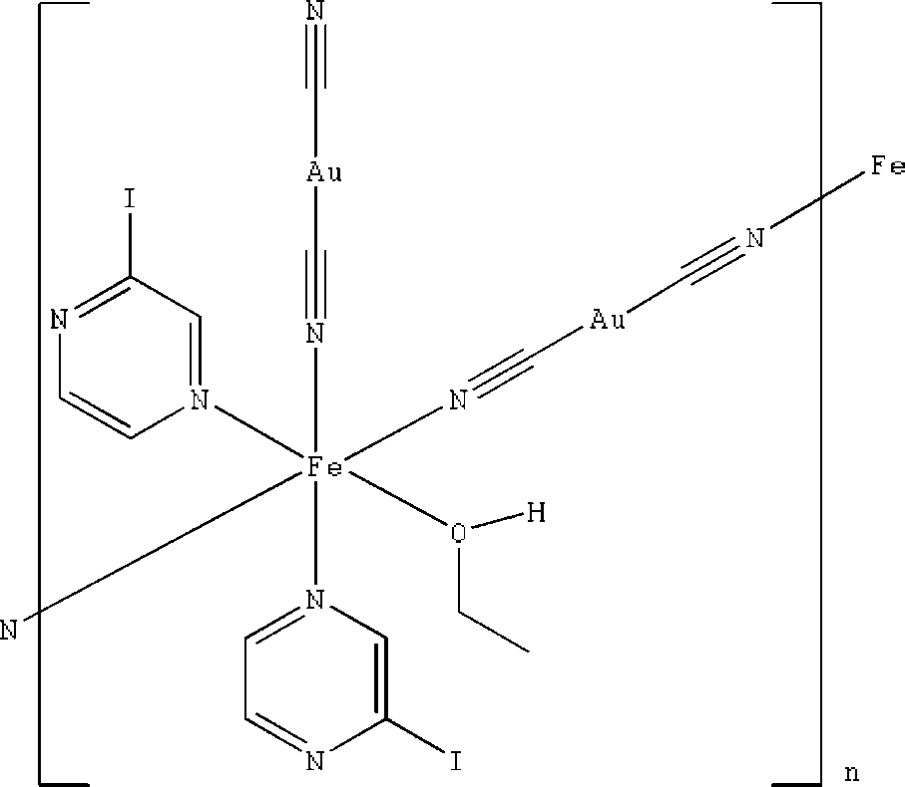


## Structural commentary   

**Figure 1:**
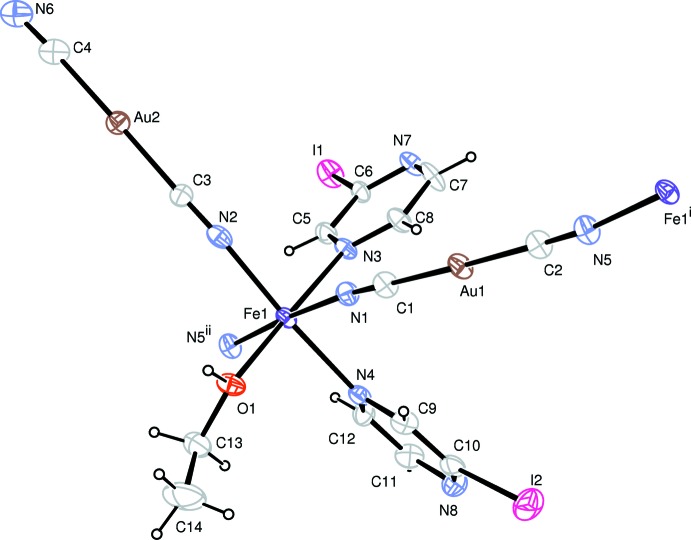
A fragment of the mol­ecular structure of the title compound showing the atom-labelling scheme. Displacement ellipsoids are drawn at the 50% probability level [symmetry codes: (i) *x*, 1 + *y*, *z*; (ii) *x*, −1 + *y*, *z*].

The crystal structure of the title compound was determined at 296 K. It crystallizes in the triclinic *P* 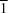 space group with two formula units per cell. The Fe^II^ site has a distorted octa­hedral [FeN_5_O] coordination environment formed by two iodo­pyrazine N atoms, three di­cyano­aurate N atoms and one ethanol O atom (Fig. 1 ▸). Two iodo­pyrazine mol­ecules are coordinated in the *cis* configuration with the Fe—N distances of 2.216 (7) and 2.272 (7) Å (Table 1 ▸) indicating the high-spin state of the Fe^II^ cation. One of the di­cyano­aurate fragments is N-coordinated to the Fe^II^ site in the form of an anion [Fe1—N2 = 2.096 (7) Å], while the other two are coordinated in a *trans* configuration, further connecting the framework into a chain [Fe1—N1 = 2.105 (8) and Fe1—N5 = 2.096 (8) Å]. The CN^−^ anions bridge the Fe^II^ and Au^I^ cations in a quasi-linear mode with C1—Au1—C2 = 178.8 (3) and C3—Au2—C4 = 178.9 (3)°. In addition, one of the coordination sites of the Fe^II^ ion is occupied by an O-coordinated ethanol molecule nwith Fe1—O1 = 2.106 (6) Å, which is a typical value for Fe—O_alcohol_ bonds. There is a deviation from an ideal octa­hedral geometry, Σ|90 - Θ| = 33.1°, where Θ is the *cis*-N—Fe—N or *cis*-O—Fe—N angle in the coordination environment of Fe^II^. This value indicates a significant polyhedral distortion that can be explained by the Jahn–Teller effect and the presence of four different types of ligands.

## Supra­molecular features   

**Figure 2:**
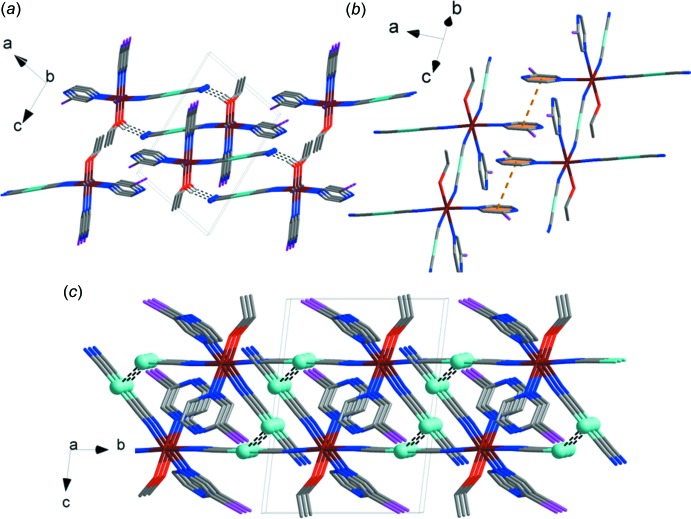
The crystal structure of the title compound. (*a*) View in the *ac* plane showing N⋯H—O hydrogen bonds (dashed lines). H atoms, except those involved in hydrogen bonding, are omitted for clarity. (*b*) Structure of the title compound showing π–π contacts (dashed lines). (*c*) View in the *bc* plane showing aurophilic inter­actions (dashed lines). Colour key: Fe dark red; Ag cyan; N blue; O red; C grey; I purple.

The coordination framework is connected by bridging di­cyano­aurate moieties into chains that propagate along the *b*-axis direction. In addition, the crystal packing is supported by N⋯H—O hydrogen bonds (Fig. 2 ▸ *a*, Table 2 ▸) in which H atoms from the ethanol hydroxyl group participate in weak inter­actions with the N atoms of the di­cyano­aurate anions. The structure includes parallel-displaced π–π inter­actions with a distance of 3.381 (5) Å between the planes of the aromatic rings (Fig. 2 ▸ *b*). Short Au ⋯ Au distances of 3.163 (5) Å indicate inter­molecular aurophilic inter­actions between the Au atoms of the monodentate and bridging di­cyano­aurate moieties (Fig. 2 ▸ *c*). The same type of aurophilic inter­action was observed for a very similar Au–Fe–pyrazine complex, which displays high-temperature spin-transition behavior [Au⋯Au (LS, 340 K) = 3.3886 (3) Å, Au⋯Au (HS, 360 K) = 3.5870 (5) Å; Gural’skiy *et al.*, 2016*a* ▸]. The Au⋯Au distances in the above-mentioned structure are longer because they are defined by a three-dimensional framework of the complex; however, in the case of the title compound, the di­cyano­aurate anions are non-bridging and therefore are more flexible, which leads to the creation of aurophilic contacts that are closer to the optimum distance of 3 Å (Schmidbaur, 2000 ▸).

## Database survey   

A survey of the Cambridge structural Database (Version 5.38; Groom *et al.*, 2016 ▸) confirmed that the title compound has never been published before. It also revealed numerous examples of CN-bridged Au–Fe bimetallic frameworks supported by substituted azines (Li *et al.*, 2015 ▸; Agustí *et al.*, 2008 ▸; Kosone *et al.*, 2009 ▸) and non-substituted azines (Niel *et al.*, 2003 ▸; Gural’skiy *et al.*, 2016*a* ▸; Kosone *et al.*, 2008 ▸).

## Synthesis and crystallization   

Crystals of the title compound were obtained by the slow-diffusion method with three layers in a 5 ml tube. The first layer was a solution of K[Au(CN)_2_] (29 mg, 0.1 mmol) in water (1 ml), the second layer was a water/ethanol mixture (1:1, 2.5 ml) and the third layer was a solution of Fe(OTs)_2_·6H_2_O (OTs = toluene­sulfonate) (50.6 mg, 0.1 mmol) and iodo­pyrazine (41.2 mg, 0.2 mmol) in ethanol (1 ml). After two weeks, yellow crystals grew in the middle layer; these were collected and kept under the mother solution prior to measurement.

## Refinement   

Crystal data, data collection and structure refinement details are summarized in Table 3 ▸. All hydrogen atoms were placed geometrically at their expected calculated positions with C—H = 0.96 (CH_3_), 0.97 (CH_2_), 0.93 Å (C_arom_), O—H = 0.859 (10) Å, and with *U*_iso_(H) = 1.2*U*_eq_(C) with the exception of methyl hydrogen atoms, which were refined with *U*_iso_(H) = 1.5*U*_eq_(C). The idealized CH_3_ group was fixed using an AFIX 137 command that allowed the H atoms to ride on the C atom and rotate around th C—C bond.

## Supplementary Material

Crystal structure: contains datablock(s) global, I. DOI: 10.1107/S2056989017014785/xu5907sup1.cif

Structure factors: contains datablock(s) I. DOI: 10.1107/S2056989017014785/xu5907Isup2.hkl

CCDC reference: 1579611

Additional supporting information:  crystallographic information; 3D view; checkCIF report

## Figures and Tables

**Table 1:** Selected bond lengths (Å)

Au1—C1	1.948 (9)	Fe1—N1	2.105 (8)
Au1—C2	1.952 (8)	Fe1—N2	2.096 (7)
Au2—C3	1.970 (8)	Fe1—N3	2.272 (7)
Au2—C4	1.981 (9)	Fe1—N4	2.216 (7)
Fe1—O1	2.106 (6)	Fe1—N5^i^	2.096 (8)

Symmetry code: (i) 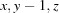.

**Table 2:** Hydrogen-bond geometry (Å, °)

*D*—H⋯*A*	*D*—H	H⋯*A*	*D*⋯*A*	*D*—H⋯*A*
O1—H1⋯N6^ii^	0.86 (5)	1.98 (5)	2.765 (13)	151 (6)

Symmetry code: (ii) 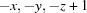.

**Table 3:** Experimental details

Crystal data	
Chemical formula	[Au_2_Fe(CN)_4_(C_4_H_3_IN_2_)_2_(C_2_H_6_O)]
*M*_r_	1011.90
Crystal system, space group	Triclinic, *P* 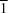
Temperature (K)	296
*a*, *b*, *c* (Å)	9.40 (2), 10.30 (2), 12.81 (3)
α, β, γ (°)	92.05 (6), 99.67 (7), 114.30 (6)
*V* (Å^3^)	1106 (4)
*Z*	2
Radiation type	Mo *K*α
μ (mm^−1^)	16.70
Crystal size (mm)	0.20 × 0.05 × 0.03

Data collection	
Diffractometer	Bruker SMART
Absorption correction	Part of the refinement model (Δ*F*) (Walker & Stuart, 1983 ▸)
*T*_min_, *T*_max_	0.298, 0.456
No. of measured, independent and observed [*I* > 2σ(*I*)] reflections	5556, 5556, 4453
*R*_int_	0.097
(sin θ/λ)_max_ (Å^−1^)	0.680

Refinement	
*R*[*F* ^2^ > 2σ(*F* ^2^)], *wR*(*F* ^2^), *S*	0.038, 0.077, 0.92
No. of reflections	5556
No. of parameters	257
No. of restraints	3
H-atom treatment	H-atom parameters constrained
Δρ_max_, Δρ_min_ (e Å^−3^)	2.26, −2.28

Computer programs: *SMART* and *SAINT* (Bruker, 2013 ▸), *SHELXT* (Sheldrick, 2015*a* ▸), *SHELXL* (Sheldrick, 2015*b* ▸), *DIAMOND* (Brandenburg *et al.*, 1999 ▸) and *OLEX2* (Dolomanov *et al.*, 2009 ▸).

## supplementary crystallographic information

### Crystal data

**Table:**

[Au_2_Fe(CN)_4_(C_4_H_3_IN_2_)_2_(C_2_H_6_O)]	*Z* = 2
*M**_r_* = 1011.90	*F*(000) = 900
Triclinic, *P*1	*D*_x_ = 3.039 Mg m^−^^3^
*a* = 9.40 (2) Å	Mo *K*α radiation, λ = 0.71073 Å
*b* = 10.30 (2) Å	Cell parameters from 5517 reflections
*c* = 12.81 (3) Å	θ = 2.4–28.1°
α = 92.05 (6)°	µ = 16.70 mm^−^^1^
β = 99.67 (7)°	*T* = 296 K
γ = 114.30 (6)°	Needle, yellow
*V* = 1106 (4) Å^3^	0.20 × 0.05 × 0.03 mm

### Data collection

**Table:**

Bruker SMART diffractometer	4453 reflections with *I* > 2σ(*I*)
φ and ω scans	*R*_int_ = 0.097
Absorption correction: part of the refinement model (Δ*F*) (Walker & Stuart, 1983)	θ_max_ = 28.9°, θ_min_ = 1.6°
*T*_min_ = 0.298, *T*_max_ = 0.456	*h* = −10→12
5556 measured reflections	*k* = −13→8
5556 independent reflections	*l* = −16→17

### Refinement

**Table:**

Refinement on *F*^2^	Primary atom site location: structure-invariant direct methods
Least-squares matrix: full	Hydrogen site location: mixed
*R*[*F*^2^ > 2σ(*F*^2^)] = 0.038	H-atom parameters constrained
*wR*(*F*^2^) = 0.077	*w* = 1/[σ^2^(*F*_o_^2^) + (0.0259*P*)^2^] where *P* = (*F*_o_^2^ + 2*F*_c_^2^)/3
*S* = 0.92	(Δ/σ)_max_ = 0.001
5556 reflections	Δρ_max_ = 2.26 e Å^−^^3^
257 parameters	Δρ_min_ = −2.28 e Å^−^^3^
3 restraints	

### Special details

**Table:**

Geometry. All esds (except the esd in the dihedral angle between two l.s. planes) are estimated using the full covariance matrix. The cell esds are taken into account individually in the estimation of esds in distances, angles and torsion angles; correlations between esds in cell parameters are only used when they are defined by crystal symmetry. An approximate (isotropic) treatment of cell esds is used for estimating esds involving l.s. planes.

### Fractional atomic coordinates and isotropic or equivalent isotropic displacement parameters (Å^2^)

**Table:**

	*x*	*y*	*z*	*U*_iso_*/*U*_eq_	
Au1	0.59034 (4)	0.85190 (3)	0.70913 (2)	0.01892 (7)	
Au2	0.11598 (3)	0.01716 (3)	0.40500 (2)	0.02116 (8)	
I1	0.85042 (7)	0.17524 (6)	0.33540 (4)	0.03085 (13)	
I2	1.09742 (8)	0.86712 (6)	0.99527 (4)	0.03868 (15)	
Fe1	0.58906 (12)	0.35008 (10)	0.69926 (7)	0.0154 (2)	
O1	0.4739 (6)	0.3148 (6)	0.8300 (4)	0.0229 (11)	
H1	0.379 (4)	0.310 (7)	0.825 (3)	0.034*	
N4	0.8280 (7)	0.4651 (6)	0.8031 (4)	0.0197 (13)	
N6	−0.1459 (9)	−0.2072 (8)	0.2275 (5)	0.0306 (16)	
N2	0.3802 (7)	0.2347 (7)	0.5851 (4)	0.0206 (13)	
N3	0.7137 (7)	0.3977 (6)	0.5585 (4)	0.0184 (12)	
N7	0.8521 (8)	0.4547 (7)	0.3799 (5)	0.0252 (14)	
C4	−0.0500 (9)	−0.1237 (9)	0.2898 (5)	0.0242 (16)	
N8	1.1356 (8)	0.6149 (7)	0.9205 (5)	0.0270 (14)	
N1	0.5698 (7)	0.5466 (6)	0.6996 (4)	0.0200 (12)	
C3	0.2821 (9)	0.1544 (8)	0.5205 (5)	0.0186 (14)	
N5	0.6142 (8)	1.1576 (7)	0.7088 (4)	0.0215 (13)	
C6	0.8096 (9)	0.3307 (8)	0.4158 (5)	0.0196 (14)	
C5	0.7384 (9)	0.2989 (8)	0.5038 (5)	0.0215 (15)	
H5	0.707513	0.207434	0.525019	0.026*	
C9	0.8752 (10)	0.5910 (8)	0.8594 (5)	0.0245 (16)	
H9	0.802733	0.630763	0.860960	0.029*	
C2	0.6042 (9)	1.0464 (8)	0.7107 (5)	0.0195 (14)	
C8	0.7562 (9)	0.5225 (8)	0.5231 (5)	0.0223 (15)	
H8	0.740253	0.594317	0.558402	0.027*	
C1	0.5725 (9)	0.6564 (8)	0.7050 (5)	0.0211 (15)	
C10	1.0299 (10)	0.6648 (8)	0.9160 (5)	0.0270 (18)	
C7	0.8257 (10)	0.5510 (9)	0.4327 (6)	0.0289 (18)	
H7	0.853931	0.641261	0.409642	0.035*	
C12	0.9357 (9)	0.4135 (8)	0.8064 (5)	0.0227 (15)	
H12	0.908739	0.325149	0.768484	0.027*	
C11	1.0847 (10)	0.4878 (9)	0.8641 (6)	0.0271 (17)	
H11	1.156693	0.447104	0.864451	0.033*	
C13	0.4932 (10)	0.2326 (9)	0.9133 (5)	0.0290 (18)	
H13A	0.603756	0.247389	0.930592	0.035*	
H13B	0.428430	0.131598	0.889179	0.035*	
C14	0.4467 (14)	0.2727 (13)	1.0106 (6)	0.055 (3)	
H14A	0.340589	0.266687	0.992360	0.082*	
H14B	0.519379	0.369119	1.039883	0.082*	
H14C	0.449934	0.208333	1.062348	0.082*	

### Atomic displacement parameters (Å^2^)

**Table:**

	*U*^11^	*U*^22^	*U*^33^	*U*^12^	*U*^13^	*U*^23^
Au1	0.02856 (16)	0.01474 (13)	0.02017 (13)	0.01414 (12)	0.00870 (11)	0.00444 (10)
Au2	0.02192 (16)	0.02330 (14)	0.01917 (13)	0.00997 (12)	0.00570 (11)	0.00122 (11)
I1	0.0417 (3)	0.0313 (3)	0.0298 (2)	0.0205 (3)	0.0201 (2)	0.0053 (2)
I2	0.0482 (4)	0.0259 (3)	0.0367 (3)	0.0140 (3)	0.0020 (3)	−0.0082 (2)
Fe1	0.0220 (5)	0.0131 (4)	0.0152 (4)	0.0100 (4)	0.0070 (4)	0.0039 (4)
O1	0.026 (3)	0.032 (3)	0.018 (2)	0.018 (3)	0.008 (2)	0.007 (2)
N4	0.026 (3)	0.019 (3)	0.017 (3)	0.011 (3)	0.005 (2)	0.007 (2)
N6	0.033 (4)	0.044 (4)	0.025 (3)	0.024 (4)	0.010 (3)	0.003 (3)
N2	0.025 (4)	0.024 (3)	0.022 (3)	0.017 (3)	0.010 (3)	0.010 (3)
N3	0.021 (3)	0.022 (3)	0.017 (3)	0.012 (3)	0.010 (2)	0.008 (2)
N7	0.034 (4)	0.025 (3)	0.025 (3)	0.018 (3)	0.013 (3)	0.013 (3)
C4	0.027 (4)	0.036 (4)	0.023 (3)	0.025 (4)	0.010 (3)	0.001 (3)
N8	0.024 (4)	0.028 (3)	0.029 (3)	0.011 (3)	0.004 (3)	0.002 (3)
N1	0.023 (3)	0.017 (3)	0.022 (3)	0.010 (3)	0.004 (2)	0.004 (2)
C3	0.019 (4)	0.019 (3)	0.022 (3)	0.011 (3)	0.003 (3)	0.003 (3)
N5	0.025 (3)	0.020 (3)	0.023 (3)	0.011 (3)	0.011 (3)	0.004 (2)
C6	0.019 (4)	0.021 (3)	0.019 (3)	0.007 (3)	0.009 (3)	0.006 (3)
C5	0.025 (4)	0.022 (4)	0.018 (3)	0.009 (3)	0.008 (3)	0.004 (3)
C9	0.031 (4)	0.027 (4)	0.022 (3)	0.017 (4)	0.007 (3)	0.003 (3)
C2	0.023 (4)	0.020 (3)	0.023 (3)	0.014 (3)	0.010 (3)	0.002 (3)
C8	0.029 (4)	0.022 (4)	0.024 (3)	0.016 (3)	0.012 (3)	0.009 (3)
C1	0.024 (4)	0.021 (4)	0.023 (3)	0.013 (3)	0.008 (3)	0.002 (3)
C10	0.041 (5)	0.024 (4)	0.014 (3)	0.010 (4)	0.011 (3)	0.002 (3)
C7	0.040 (5)	0.026 (4)	0.032 (4)	0.018 (4)	0.024 (4)	0.019 (3)
C12	0.026 (4)	0.018 (3)	0.024 (3)	0.009 (3)	0.005 (3)	0.001 (3)
C11	0.030 (4)	0.033 (4)	0.026 (4)	0.021 (4)	0.005 (3)	0.004 (3)
C13	0.030 (5)	0.035 (4)	0.025 (4)	0.015 (4)	0.009 (3)	0.010 (3)
C14	0.060 (7)	0.086 (8)	0.024 (4)	0.032 (7)	0.017 (4)	0.011 (5)

### Geometric parameters (Å, º)

**Table:**

Au1—Au2^i^	3.163 (5)	N7—C7	1.309 (10)
Au1—C1	1.948 (9)	N8—C10	1.287 (11)
Au1—C2	1.952 (8)	N8—C11	1.329 (10)
Au2—C3	1.970 (8)	N1—C1	1.119 (10)
Au2—C4	1.981 (9)	N5—C2	1.111 (10)
I1—C6	2.071 (8)	C6—C5	1.387 (9)
I2—C10	2.078 (9)	C5—H5	0.9300
Fe1—O1	2.106 (6)	C9—H9	0.9300
Fe1—N1	2.105 (8)	C9—C10	1.384 (11)
Fe1—N2	2.096 (7)	C8—H8	0.9300
Fe1—N3	2.272 (7)	C8—C7	1.404 (10)
Fe1—N4	2.216 (7)	C7—H7	0.9300
Fe1—N5^ii^	2.096 (8)	C12—H12	0.9300
O1—H1	0.859 (10)	C12—C11	1.350 (11)
O1—C13	1.419 (9)	C11—H11	0.9300
N4—C9	1.323 (10)	C13—H13A	0.9700
N4—C12	1.318 (10)	C13—H13B	0.9700
N6—C4	1.122 (10)	C13—C14	1.486 (11)
N2—C3	1.134 (9)	C14—H14A	0.9600
N3—C5	1.333 (9)	C14—H14B	0.9600
N3—C8	1.302 (9)	C14—H14C	0.9600
N7—C6	1.298 (9)		
			
C2—Au1—Au2^i^	82.5 (2)	N7—C6—C5	123.1 (7)
C1—Au1—Au2^i^	97.8 (2)	C5—C6—I1	119.6 (5)
C1—Au1—C2	178.8 (3)	N3—C5—C6	120.9 (7)
C4—Au2—Au1^i^	101.9 (3)	N3—C5—H5	119.5
C3—Au2—Au1^i^	78.1 (3)	C6—C5—H5	119.5
C3—Au2—C4	178.9 (3)	N4—C9—H9	119.3
O1—Fe1—N4	92.6 (3)	N4—C9—C10	121.4 (8)
O1—Fe1—N3	177.6 (2)	C10—C9—H9	119.3
N4—Fe1—N3	87.1 (3)	N5—C2—Au1	177.8 (7)
N2—Fe1—O1	94.9 (3)	N3—C8—H8	119.3
N2—Fe1—N4	171.7 (2)	N3—C8—C7	121.5 (7)
N2—Fe1—N3	85.6 (3)	C7—C8—H8	119.3
N2—Fe1—N1	95.8 (3)	N1—C1—Au1	175.8 (7)
N1—Fe1—O1	86.5 (2)	N8—C10—I2	118.1 (6)
N1—Fe1—N4	88.1 (3)	N8—C10—C9	123.0 (7)
N1—Fe1—N3	91.1 (2)	C9—C10—I2	118.9 (6)
N5^ii^—Fe1—O1	91.3 (2)	N7—C7—C8	122.2 (7)
N5^ii^—Fe1—N4	89.4 (3)	N7—C7—H7	118.9
N5^ii^—Fe1—N2	87.0 (3)	C8—C7—H7	118.9
N5^ii^—Fe1—N3	91.1 (2)	N4—C12—H12	119.7
N5^ii^—Fe1—N1	176.6 (2)	N4—C12—C11	120.6 (7)
Fe1—O1—H1	122.0 (18)	C11—C12—H12	119.7
C13—O1—Fe1	126.0 (5)	N8—C11—C12	124.5 (8)
C13—O1—H1	105.7 (18)	N8—C11—H11	117.8
C9—N4—Fe1	123.4 (5)	C12—C11—H11	117.8
C12—N4—Fe1	120.4 (5)	O1—C13—H13A	109.3
C12—N4—C9	116.1 (7)	O1—C13—H13B	109.3
C3—N2—Fe1	165.9 (6)	O1—C13—C14	111.7 (8)
C5—N3—Fe1	122.6 (5)	H13A—C13—H13B	107.9
C8—N3—Fe1	120.9 (5)	C14—C13—H13A	109.3
C8—N3—C5	116.4 (6)	C14—C13—H13B	109.3
C6—N7—C7	116.0 (6)	C13—C14—H14A	109.5
N6—C4—Au2	177.2 (7)	C13—C14—H14B	109.5
C10—N8—C11	114.3 (7)	C13—C14—H14C	109.5
C1—N1—Fe1	174.0 (6)	H14A—C14—H14B	109.5
N2—C3—Au2	178.2 (6)	H14A—C14—H14C	109.5
C2—N5—Fe1^iii^	169.9 (7)	H14B—C14—H14C	109.5
N7—C6—I1	117.3 (5)		
			
I1—C6—C5—N3	178.6 (5)	C6—N7—C7—C8	0.3 (12)
Fe1—O1—C13—C14	158.4 (6)	C5—N3—C8—C7	−0.6 (11)
Fe1—N4—C9—C10	−174.4 (5)	C9—N4—C12—C11	−0.3 (10)
Fe1—N4—C12—C11	175.7 (5)	C8—N3—C5—C6	1.6 (10)
Fe1—N3—C5—C6	177.5 (5)	C10—N8—C11—C12	−0.3 (11)
Fe1—N3—C8—C7	−176.6 (6)	C7—N7—C6—I1	−179.6 (6)
N4—C9—C10—I2	176.5 (5)	C7—N7—C6—C5	0.8 (11)
N4—C9—C10—N8	−2.2 (11)	C12—N4—C9—C10	1.5 (10)
N4—C12—C11—N8	−0.3 (12)	C11—N8—C10—I2	−177.3 (5)
N3—C8—C7—N7	−0.4 (13)	C11—N8—C10—C9	1.5 (10)
N7—C6—C5—N3	−1.8 (11)		

Symmetry codes: (i) −*x*+1, −*y*+1, −*z*+1; (ii) *x*, *y*−1, *z*; (iii) *x*, *y*+1, *z*.

### Hydrogen-bond geometry (Å, º)

**Table:**

*D*—H···*A*	*D*—H	H···*A*	*D*···*A*	*D*—H···*A*
O1—H1···N6^iv^	0.86 (5)	1.98 (5)	2.765 (13)	151 (6)

Symmetry code: (iv) −*x*, −*y*, −*z*+1.
